# Refining Host-Pathogen Interactions: Organ-on-Chip Side of the Coin

**DOI:** 10.3390/pathogens10020203

**Published:** 2021-02-13

**Authors:** Buket Baddal, Pasquale Marrazzo

**Affiliations:** 1 Department of Medical Microbiology and Clinical Microbiology, Faculty of Medicine, Near East University, Nicosia 99138, Cyprus; 2 Department of Experimental, Diagnostic and Specialty Medicine, Alma Mater Studiorum University of Bologna, 40126 Bologna, Italy; pasquale.marrazzo2@unibo.it

**Keywords:** microfluidics, organ-on-chip, host-microbe interactions, infectious disease, therapeutics

## Abstract

Bioinspired organ-level in vitro platforms that recapitulate human organ physiology and organ-specific responses have emerged as effective technologies for infectious disease research, drug discovery, and personalized medicine. A major challenge in tissue engineering for infectious diseases has been the reconstruction of the dynamic 3D microenvironment reflecting the architectural and functional complexity of the human body in order to more accurately model the initiation and progression of host–microbe interactions. By bridging the gap between in vitro experimental models and human pathophysiology and providing alternatives for animal models, organ-on-chip microfluidic devices have so far been implemented in multiple research areas, contributing to major advances in the field. Given the emergence of the recent pandemic, plug-and-play organ chips may hold the key for tackling an unmet clinical need in the development of effective therapeutic strategies. In this review, latest studies harnessing organ-on-chip platforms to unravel host–pathogen interactions are presented to highlight the prospects for the microfluidic technology in infectious diseases research.

## 1. Introduction

Throughout the timeline of human tissue models, major breakthroughs and convergence from simple two-dimensional (2D) tissue cultures to complex humanoid microfluidic systems have been accomplished in an effort to replicate human physiology, recreate functions of organs, and predict immune and drug responses. The concept of simulating human organ-level functions and disease using a microfluidic chip was first described in 2004 [1,2], and the term organ-on-chip was consequently coined [3]. Since then, a plethora of studies have focused on the development of microfluidic devices that mimic diverse biological functions of airways [4,5], gut [6,7], liver [8,9], kidney [10], brain/blood-brain barrier [11,12], heart [13], skin [14], blood vessels [15], bones [16], adipose tissues [17], placenta [18], and diseases such as cancer [19,20], thrombosis [21], Alzheimer’s disease [22], and infection [23]. These mimetic devices serve as human surrogates for the prediction of clinical outcomes by exhibiting hallmarks of native tissues, including critical tissue–tissue interfaces, spatiotemporal chemical gradients, and dynamic mechanical cues of organ microenvironments such as contractile properties, cyclic breathing, blood flow, shear force, cell patterning, tissue barrier function, circulating immune cell recruitment, as well as integration of the complex microbiome—which collectively offer unprecedented capabilities over conventional in vitro models [24,25]. Systematic interaction of multiple organs such as drug absorption, distribution, metabolism, and elimination involving the gut, circulation, liver, and kidney is one of the primary focuses of organ-on-chip (also known as organ chip) technology. Microfluidics techniques such as soft-lithography, replica molding, and, more recently, advanced techniques such as three-dimensional (3D) printing have been employed for the biofabrication of microfluidic devices and have been thoroughly reviewed from a developmental and applications perspective previously [26,27,28].

**Figure 1:**
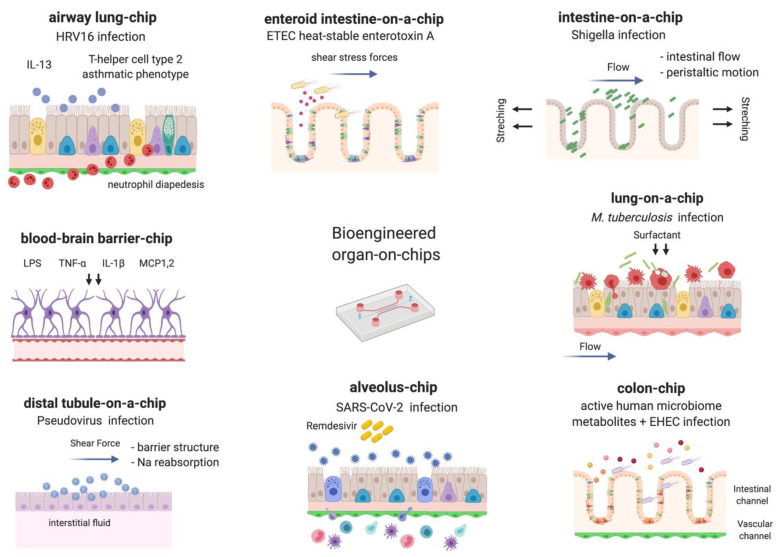
Current applications of organ chips in infectious diseases.

Modeling of the native tissue microenvironment and mucosal surfaces lining the respiratory, gastrointestinal, and urogenital tracts for infectious diseases have been attempted in a broad range of models ranging from reductionist flat 2D models to rotating wall vessel bioreactors, extracellular matrix (ECM)-embedded 3D cultures, organoids, and organs-on-chip [29]. Organ chip platforms have enabled the exploration of complex pathophysiological features of human microbial infections, including pathogenic and nonpathogenic commensal bacteria; viral, fungal and parasitic microorganisms; stages of infection such as colonization, internalization, invasion, and biofilm formation; cross talk between epithelium and endothelium; evasion from the host immune response and quantification of immune cell responses; mimicry of clinical responses to antimicrobial therapeutic agents; and spontaneous microbial evolution in response to drug exposure. With the growing demand of the unforeseen coronavirus disease 2019 (COVID-19) pandemic due to recently emerged severe acute respiratory syndrome coronavirus 2 (SARS-CoV-2), organ chips have been at the frontier of modeling human lung infections to elucidate complex mechanisms involved in viral pathogenesis. They have driven the re-purposing of existing Food and Drug Administration (FDA)-approved drugs, accelerated drug discovery and monitoring of novel compounds [30] (Figure 1). This review describes the latest advances within organ-on-chip platforms in the context of host–pathogen interactions.

## 2. Bacteria

With the current understanding of infection biology, microorganisms play a central role in shaping human physiology, which has triggered interest in the field of host–microbe interactions. A particularly confounding issue of animal or standard in vitro models has been the inability to culture certain pathogens due to the lack of pathogen-specific cell types or cellular receptors. Besides, the failure to recapitulate the human response to infection and the inability to implement manipulations in real-time or perform live observations/imaging of transient events at the microscopic level have been among the shortcomings of traditional models [31]. 

The intracellular lifestyle of *Shigella* as an enteric pathogen inducing bacillary dysentery in the human colon has been extensively studied. However, interrogation of *Shigella* invasion mechanisms within the mucosal epithelium, which acts as a gatekeeper against infectious microorganisms, has been largely limited. This is mainly due to the notorious inefficiency of the bacterium to infect polarized epithelial monolayers at the apical surface, both in Transwell systems [32] and more recently in human colorectal biopsy-derived enteroids [33,34]. So far, a range of gut chips emulating different regions of the human intestine have been developed based on anatomical and physiological functions [35,36,37]. By incorporating two main physical forces naturally imposed by intestinal epithelium on colonizing pathogens—shear stress by intestinal flow and tensile forces induced by stretching of the underlying muscle layer leading to peristalsis—Grassart and colleagues reconstructed mucosal tissue colonization by *Shigella flexneri* in the intestine chip [38]. Using this innovative approach, the ability of the pathogen to invade enterocytes from the apical side and the triggering of the loss of barrier integrity was demonstrated. Furthermore, the impact of peristalsis-like mechanical deformations on the increased capability of the bacterium to invade human colonic epithelium was discovered, which would have been overlooked in conventional models with oversimplified epithelial microenvironment. Excitingly, the effect of mechanical stimuli was also recently highlighted for enterotoxigenic *Escherichia coli* (ETEC) infection, which is associated with a high burden of diarrheal diseases worldwide. Sunuwar et al. utilized an intestine chip comprised of human jejunal enteroids in order to assess the effects of flow and repetitive stretch on the secretion of cyclic GMP (cGMP) in response to ETEC heat-stable enterotoxin A (ST). In this study, the authors were able to show, for the first time, that luminal flow and serosal blood flow both enhance secretion of cGMP upon ST exposure, which ultimately leads to intestinal fluid and electrolyte loss [39].

Organ-on-chip technology has also contributed to our understanding of infections by respiratory pathogens. The host-protective role of pulmonary surfactant in early tuberculosis instigated by the pathogen *Mycobacterium tuberculosis* has been described in literature. However, a complete understanding of the role of surfactant in bacterial infection could not be established due to the lethality of surfactant deficiency in animal models [40]. Thacker et al. used lung-on-chip device to create a tailored model of early *M. tuberculosis* infection with alveolar epithelial cells and macrophages, including channels that mimic air and blood flow as well as surfactants. By measuring the growth of intracellular bacteria with and without surfactant via time-lapse imaging, the authors were able to define a mechanism in which pulmonary surfactants led to the removal of virulence-associated lipids from *M. tuberculosis* cell surface, thereby attenuating bacterial intracellular growth in macrophages [41]. Furthermore, organ chips were proposed as a promising platform for mechanistic studies of host–pathogen interaction in the context of co-infection. Bronchopneumonia, a major infectious disease of the lower respiratory tract, is often caused by the seasonal influenza virus. In such infections, a high mortality is commonly observed due to onset of a subsequent bacterial superinfection. Deinhardt-Emmer et al. established a co-infection model using alveolus-on-chip composed of vascular and epithelial cell structures with co-cultured tissue-resident macrophages resembling the human alveolus architecture and function [42]. Via monitoring of spatiotemporal spread of the pathogens, the authors revealed a significant impairment of endothelial barrier integrity, higher inflammatory response, and spread to the endothelium during co-infection, compared to a single infection of the epithelium. This alveolus model was also proposed for the study of human-specific bacterial toxins such as Panton–Valentine leukocidin that target immune cells, hence broadening the horizons for further investigation on viral–bacterial co-infections in which neutrophil granulocytes play a central role.

**Figure 2:**
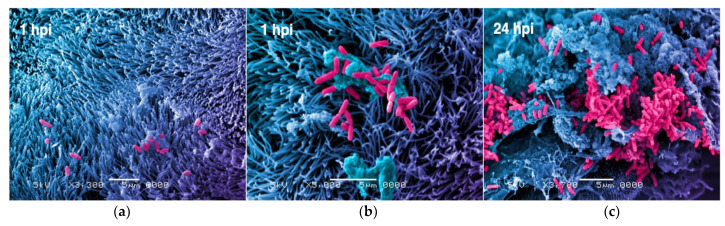
Tracking of host colonization and stages of biofilm formation by *Pseudomonas aeruginosa* on human small airway chip. (**a**) Airway infection at 1 h post infection as bacteria attach to airway mucociliary epithelium; (**b**) bacterial cells trapped in mucus at initial attachment on ciliated epithelial cells; (**c**) fully developed biofilm with exopolysaccharide matrix at 24 h is shown (used with permission from Emulate, Inc.).

An important step in bacterial pathogenesis with therapeutic implications is biofilm formation. Mucosal attachment, colonization, microcolony formation, and biofilm maturation and dispersal are among the stages of biofilm development that can be mimicked in vitro for drug screening purposes. Figure 2 demonstrates steps of biofilm formation by *Pseudomonas aeruginosa* on human mucociliary bronchiolar epithelium in an airway chip (unpublished data). Although organ-chip models such as skin-on-a-chip have been widely used for pharmacology, toxicology, and regenerative applications, the application of these systems in skin modeling of microbial infections and biofilms to investigate their implications in chronic infections and wound-healing processes by inducing inflammation remains limited [43]. Although lacking human cells, in vitro microfluidic wound models have been developed to examine *Staphylococcus pseudointermedius* biofilms under antibiotic treatment [44], as well as complex polymicrobial biofilms of *P. aeruginosa* and *E. coli* [45].

## 3. Viruses

Infection kinetics, virus–host interactions, viral evolution, and emergence of drug resistance represent some of the prospects of microfluidics technology that spurred the use of organ chips for modeling viral diseases [46]. Benam et al. in 2015 engineered a human lung small airway-on-a-chip that supports full differentiation of columnar, pseudostratified, mucociliary bronchiolar epithelium of healthy individuals as well people with chronic obstructive pulmonary disease. Using this model recapitulating the lung microenvironment, the authors simulated mucosal inflammation and exacerbations by viral pathogens by exposing the airway epithelium to viral mimic polyinosinic-polycytidylic acid, an analogue of double-stranded RNA produced in infected cells during viral replication by respiratory viruses. Via this approach, they demonstrated a robust model of human lung inflammatory disorders with disease features including selective cytokine hypersecretion, increased neutrophil recruitment, and clinical exacerbations [47]. 

Defining host–pathogen interactions in viral hepatitis and associated liver disease has so far been limited by the narrow host range of the hepatitis B virus (HBV) and the inability to reproduce the complexity of the liver environment using conventional methods [48]. The application of organ chips to HBV research began with a rat liver sinusoid chip system to study viral replication [49], which was followed by a human source model via incorporation of primary human hepatocytes (PHHs) and reported expression of hepatitis B core antigen (HBcAg) as well as cellular secretion of HBV DNA post HBV infection [50]. In improved liver chip models, PHHs were cultured alone or in combination with Kupffer cells, which exhibited higher susceptibility to HBV infection compared to 2D cultures or liver spheroids. This particular model enabled the recapitulation of all steps of the HBV life cycle, including the replication of patient-derived HBV and the maintenance of HBV covalently closed circular DNA (cccDNA) [51]. 

Another challenging aspect of infectious diseases to mimic in vitro is viral infection of the gastrointestinal (GI) tract. Limitations of animal models in this respect, such as the lack of virus-associated specific cell surface receptors for infection or bypassing of the native primary infection site in humans by the inoculated virus, primed the use of microfluidic humanized chip models for viral infections of the gut. A particular example was the coxsackievirus B1 infection in a gut chip model. Using a dynamic gut-chip prototype, Villenave et al. demonstrated that human enterovirus infection, replication, and infectious virus production can be analyzed in vitro with highly differentiated human villus intestinal epithelium under conditions of fluid flow and peristalsis-like motions [52]. This study broadened the possibility of investigating mechanisms of enterovirus pathogenesis using organomimetic devices.

The nervous system—composed of a collection of distinctive types of nerves and neurons, extensive vasculature, and somatic structural support—is defined as a highly complex system in the human body. Due to evolutionary diversity, animal models cannot faithfully replicate neuron development and disease progression in humans. Moreover, the majority of in vitro experimental models of nervous system diseases suffer from the lack of cell–cell contact and interstitial fluid flow, which ultimately affect nonsynaptic neuron communication. In order to circumvent these issues, bioinspired organ-chip technology has been explored and facilitated the deciphering of pathological and physiological processes in neuronal networks [53]. Johnson et al. recently introduced a 3D printed nervous system-on-a-chip that can be loaded with central nervous system (CNS) and peripheral nervous system (PNS) neurons as well as Schwann cells for the study of viral infections. With this micro-extrusion 3D printing technology, the authors enabled the assembly of scaffold components for the alignment of axonal networks and spatial organization of cellular components. By infusion of pseudorabies virus (PRV)-infected PNS neurons into the organ chip, the authors were able to track viral replication and axon-to-cell spread of viral particles for the first time [54]. Ultimately, human kidney models have been developed to replicate the microenvironment of distal renal tubules. Renal dysfunctions are known to occur in viral infections, and multiple viruses particularly infect distal renal tubules. In distal tubule-on-a-chip, Madin Darby canine kidney (MDCK) cells were cultured on porous membranes creating transcellular transport into the static well, which reproduced an interstitial fluid environment. This model was exploited for the study of PRV-induced kidney dysfunction pathogenesis. Upon infection of the model with PRV, the reabsorption barrier was reported to lose integrity with transformed apical microvilli, and renal electrolyte dysregulation was observed with reduced Na^+^ reabsorption, all of which demonstrate virus-related pathogenesis [55].

More recently, microengineered airway chips were used to model key features of human rhinovirus 16 (HRV16)-induced exacerbations of asthma, and simultaneously assess the efficacy of immune modulators such as CXR2 antagonist during infection [56]. Furthermore, airway chips were employed to study viral replication by different strains of influenza virus, H1N1, H3N2, and H5N, responsible for outbreaks. These models rendered the quantification of host cytokine and immune cell responses to each strain relevant to the in vivo human response. Strikingly, in their study, Si et al. utilized a novel approach in which airway chips were infected with H1N1 and viruses were passaged from chip to chip eight times under the selection pressure of clinically used anti-influenza drugs amantadine and oseltamivir. Sequencing of viral genome post-passaging revealed a pool of drug-resistant strains for which mutations were described in clinical cases [57]. Additionally, in the same study, the airway chip was demonstrated to support viral evolution through gene assortment via co-infecting single chips with H3N1 and H1N1 viruses, another phenomenon that naturally occurs when different viral strains co-infect the same host [58]. 

With the rising threat of the COVID-19 pandemic, the need for the development of new preclinical discovery platforms—which can more rapidly identify therapeutics active in vitro and also translate in vivo—has grown. To this end, the lung-chip platform was leveraged in an effort to repurpose FDA-approved drugs as potential therapeutics against SARS-CoV-2. Following a proof-of-concept study with influenza A virus, the authors used airway chips to mimic airborne infection by SARS-CoV-2. SARS-CoV-2 pseudoparticles (SARS-CoV-2pp) carrying the viral spike protein, essential for SARS-CoV-2 receptor binding and entry into host cells, were introduced into the air channel, exposing the human lung epithelium that expressed angiotensin-converting enzyme 2 (ACE2) and transmembrane protease serine 2 (TMSPRSS2) to pseudotyped SARS-CoV-2 virus. By administering previously approved drugs in the airway chips under flow at a clinically relevant dose prior to infection, amodiaquine and toremifene were identified as potential entry inhibitors for SARS-CoV-2 [59]. Another recent work was reported by Zhang et al. who described a biomimetic human disease model of SARS-CoV-2 infection, recapitulating lung injury and immune responses induced by the virus on chip, hence providing a unique platform that closely mirrored human-relevant responses to SARS-CoV-2 infection [60]. These pioneering studies have unlocked a new step for the use of airway chips to accelerate drug discovery and drug repurposing during viral pandemics. 

A further recent application of the chip technology to epidemic viruses was Ebola virus infection. By employing a high-throughput microvessel-on-a-chip system, Junaid et al. modeled the Ebola hemorrhagic syndrome for which impairment of vascular integrity represents a hallmark of infection. The model was established with 96 perfusable endothelialized microvessels within a fabricated phase-guide channel system cultured with HUVECs at an interface of collagen type I under continuous perfusion. Mimicking of Ebola virus infection was performed by luminal infusion of Ebola virus-like particles as well as Ebola glycoprotein, which led to a substantial increase in vascular permeability and cytoskeleton remodeling. In addition, the authors measured the potency of a recently developed experimental drug FX06 and a novel drug candidate, melatonin, in phenotypic rescue and were able to demonstrate their therapeutic effects on the vasculopathy model. These advances in the field are expected to promote future studies in which biomimetic organ chips will be used as alternatives to preclinical models to study elements of viral pathogenesis and screening of viral therapeutic options [61].

## 4. Fungi and Parasites

Much of the infection biology research for parasites exploiting unrivalled organ chips has so far been focused on the malaria parasite *Plasmodium falciparum*. The human spleen is an immune sentinel that senses subtle mechanical changes in infected and uninfected red blood cells (RBCs), discriminates between them in malaria-infected subjects, and through this filtering function may regulate parasite biomass and induce clinical signs of malaria such as splenomegaly and anemia [62]. Sequestration of *P. falciparum*-infected red blood cells (IRBC) in the microcirculation represents a critical event in severe malaria pathogenesis. However, no accurate in vitro human capillary model that exhibits the geometric constraints governing IRBC flow through narrow human capillaries exists. Therefore, most studies are based on postmortem analysis, or studies in non-natural hosts, such as rat or primate models in which natural binding partners for parasitic ligands are lacking. To overcome these constraints, in 2014, the first functional microengineered model of the human spleen-on-a-chip was developed to evaluate the mechanical and physiological responses of the spleen using human RBCs and malaria-infected cells [63]. In 2018, an organ-on-chip device that mimics the lumen of a blood microvessel was described. This organ chip was used to determine the role of endothelial glycocalyx in the regulation of *P. falciparum* cytoadherence, supporting the proposed role of glycocalyx disruption in the pathogenesis of malarial disease [64]. Later in 2020, Arakawa et al. developed a robust 3D microvessel model that emulates the arteriole–capillary–venule transition. By comparing wild-type *P. falciparum*-infected RBCs to those infected with parasites lacking cytoadhesion ligands or membrane-stiffening knobs, the authors illustrated distinctive spatial and temporal kinetics of cell accumulation, linked to velocity transition in arteriole–capillary–venule. This study based on a new engineered model of human capillaries has shed light on mechanisms of microcirculatory obstruction in malaria and constitutes a novel platform for studying hematologic and microvascular diseases [65]. Although organ-level models are still limited for the study of parasitic infections, multiple different variations of chip technology that lack human cells but simulate circulation and mechanical retention of RBCs in the spleen [66], or lab-on-a-chip devices that integrate microfluidics and electrochemical detection for rapid diagnostics are also in development [67]. 

*Cryptosporidium parvum* is yet another parasitic agent that, despite its major impact on human health, remains poorly known in terms of pathogenesis mainly due to the lack of a long-term culture method. Although colonic explants have been used as in vitro models of cryptosporidiosis [68], research in *Cryptosporidium* biology is lagging behind other protozoan parasites. Many researchers in the field have indicated that microfluidic gut-on-a-chip devices may be able to provide the ideal conditions for *Cryptosporidium* growth in vitro [69] or that the integration of organoids derived from different organs on body-on-a-chip platform may sustain complex helminth life cycles [70]. However, the use of organ chip technology for this specific microorganism has not yet been successfully implemented.

In terms of fungal pathogens, the adoption of microfluidic technology has been more focused on the detection of fungal infections and pathogen identification at the point-of-care and clinical setting [71] or as drug screening systems for novel antifungal drugs [72]. Although microfluidic platforms have been developed for studying the germination process of fungal spores in *Cryptococcus neoformans* at high resolution [73], these models lacked human cells and are therefore out of the scope of the current manuscript. Yet to be developed, GI tract simulation of fungus–host microbiota interplay on organ chips for the identification of novel biomarkers, including fungal or host genetic polymorphisms, microbiota profiles, metabolites, or immune markers, as well as the modelling of the anti-fungal immunity in the human intestine using organ-chip systems are among the future prospects of fungal research.

## 5. Microbiome

Studies focusing on the microbiome have highly benefited from the advances of microfluidic organ chip technology. By providing a platform that maintains the physiological and pathophysiological responses of tissues, interaction with pathogenic microorganisms, and the cross talk with the immune cells, tissue chips enabled the inclusion of microbiome elements for a more comprehensive modeling of the human in vivo microenvironment [74]. Commensal bacteria are known to play a role in dysbiosis, disease development, and chronic disorders, therefore gaining a deeper understanding of the underlying mechanisms involved in host–microbiome cross talk remains crucial.

The GI tract, besides being a key site of immune surveillance, is also inhabited by a microbial community, the gut microbiome, which plays an important role in normal intestinal function and has been implicated in multiple intestinal diseases, including celiac disease, inflammatory bowel disease (IBD), and gastrointestinal malignancy. One of the first studies reporting the co-culture of the commensal bacterium *Lactobacillus rhamnosus* GG within the gut-on-a-chip system was described in 2012, and an improvement of the intestinal barrier function was observed as a similar phenomenon that happens with probiotic bacteria strains in humans [75,76]. A proof-of-concept study was designed by Kim et al. in 2016, in which intestinal epithelial cells were co-cultured with eight strains of beneficial probiotic bacteria (*Lactobacillus acidophilus*, *Lactobacillus plantarum*, *Lactobacillus paracasei*, *Lactobacillus delbrueckii* subsp. *bulgaricus*, *Bifidobacterium breve*, *Bifidobacterium longum*, *Bifidobacterium infantis*, and *Streptococcus salivarius* subsp. *thermophilus*). In the presence of commensal bacteria, intestinal cells displayed distinct gene expression profiles that closely resembled the normal human ileum. Notably, by removing peristalsis-like motions during luminal flow, the authors demonstrated that a lack of epithelial deformation triggers bacterial overgrowth similar to that occurring in IBD patients. In the same study, intestinal inflammation on the gut chip was triggered via the co-culture of intestinal epithelial cells with immune cells and exogenous addition of lipopolysaccharide endotoxin. This resulted in the production of four proinflammatory cytokines (interleukin (IL)-8, IL-6, IL-1β, and tumor necrosis factor (TNF)-α) inducing villus structure injury and impaired barrier function. More importantly, the authors revealed that the extent of villus injury and the compromised intestinal barrier function was reduced and delayed by the addition of commensal bacteria into the chip. This study indicated that the analysis of microbiome contributions to intestinal pathophysiology and the dissection of disease mechanisms in a controlled fashion can be possible on the human gut-on-a-chip [77].

A different approach was taken by Shin and Kim, who designed a pathomimetic “gut inflammation-on-a-chip” in which gut cells were treated with dextran sulfate (DSS) to induce inflammation leading to impairment of epithelial barrier integrity and villous microarchitecture. Within this model, the authors elegantly showed that the direct contact of DSS-sensitized epithelium and immune cells elevated oxidative stress. Stimulation by lipopolysaccharide (LPS) or nonpathogenic *E. coli* was also demonstrated to elicit production of inflammatory cytokines and immune cell recruitment. In this case, subsequent administration of probiotic treatment comprising eight strains of probiotic gut bacteria reduced oxidative stress in the gut cells but failed to rescue barrier dysfunction when administered after DSS-induced barrier disruption. This study hence proved the mechanistic contribution of barrier dysfunction to the onset of intestinal inflammation that is mediated by intercellular host-microbiome signaling [78]. 

Another major study regarding the interplay between the microbial flora and the host was described by Tovaglieri et al. in 2019. Metabolites generated by host gut microbiome have been shown to be important modulators of host pathophysiology and to confer protection against bacterial infection in animal models [79,80]. Interactions of enterohemorrhagic *E. coli* (EHEC) with the host have been commonly investigated in mice. However, there is an intrinsic species–specific difference in EHEC infection that limits translation of murine research to human. By simulating EHEC infection in a human colonic epithelium gut chip, the authors were able to compare the effect of metabolites derived from human gut microbiome and mouse gut microbiome and discovered that the epithelial cell injury was higher when the cells were exposed to human microbiome-derived metabolites. Furthermore, via a multi-omics approach, the authors discovered four human microbiome metabolites that induced the expression of flagellin, a bacterial protein associated with motility. Overall, results of this study indicated a possible link between the human intestinal microbiome and tolerance to infection [81].

Understanding of the relationship between human nutrition and human microbiota is an additional relevant application of microbiota gut-on-chip systems. The concept of NutriChip, an integrated microfluidic platform for the investigation of immune cell responses to pro-inflammatory stimuli and immunomodulators in food, was introduced by Ramadan et al. NutriChip was designed as a miniaturized artificial human GI, consisting of upper chamber with confluent monolayer of epithelial cells that interacted with macrophages in the lower chamber through a permeable membrane and formed the basis of future studies offering insights into gut microbiota and nutrition [82]. The human–microbial cross-talk (HuMix) model, another well designed modular microbiota-gut-on-chip system, was presented by Shah et al, which endorsed the co-culture of human and microbial cells under conditions representative of the GI human–microbe interface. Using HuMix, the authors successfully co-cultured human intestinal epithelial cells with either the facultative anaerobe *Lactobacillus rhamnosus* GG (LGG) only, or in combination with the obligate anaerobe *Bacteroides caccae* under anaerobic conditions. A detailed molecular analysis revealed that co-culture with both microorganisms resulted in a distinct transcriptional response different to the co-culture comprising only LGG. This study further elaborated the facilitation of host–microbe molecular interactions and the link between GI microbiome with health and disease in humans [83]. A comprehensive review on next generation gut-microbiota-on-chips has recently been available in literature [84].

## 6. Moving Forward: What’s Next on the Chip?

Applications of organ chips are inevitably expanding to a wider spectrum of infectious diseases and antimicrobial discovery research by overcoming the technical barriers of existing technologies. A particularly remarkable approach was the use of mucus-producing lung-on-chip to investigate the trilateral interactions among bacteria, bacteriophages (phages), and mucosal epithelium. By employing a microfluidic model that emulated a mucosal surface experiencing constant fluid flow and mucin secretion dynamics, Barr et al. analyzed phage adherence to mucus layer, thereby establishing a non-host derived layer of immunity against bacterial infections [85]. While phages have been explored thoroughly for their antimicrobial properties, most of the therapeutic validation has been done in animal models. Organ-on-chip platforms are offering unprecedented grounds for the validation of phage therapy approaches within organ of interest, with the possibility of tracking the emergence of phage resistance. Moreover, gut chips may provide a more credible microenvironment for the study of gut phageome, enabling phage-bacteria experimental studies as well as combinatory approaches to track phage adaptation, such as rapid evolution and adaptation of phage-encoded immunoglobulin-like domains within the human gut [86]. Chip devices are also amenable to the introduction of genetically modified phages and bacteria for real-time tracking via insertion of fluorescence markers or CRISPR locus for the quantification of target populations, hence rendering them valuable tools for evolutionary science.

## 7. Limitations

As with every emerging technology, the organ-on-chip platform comes with its challenges. It is important to note that organ chips may exhibit considerable variation and inconsistency between different manufactured batches, different laboratories, as well as different users [87]. From a bioengineering point of view, the biomaterials used can also have effects on the performance of the manufactured chips. While polydimethylsiloxane (PDMS) is the most commonly preferred material due to its high biocompatibility, transparency, and oxygen permeability, high level adsorption of proteins on the surface of PDMS have been reported by multiple studies, which results in the given drug or stimulating substance to not to fully interact with the cells within the chip [88]. In addition, the interaction between the cells and ECM is crucial for organ function as well as pathogen colonization; however, synthetic biomaterials usually possess mechanical properties different to native ECM [89]. While patient-specificity is a remarkable aspect of organ chips, patient tissue cells are usually limited in number or exhibit low proliferation, and their collection may require invasive techniques. The time factor has also been indicated as a potential limiting factor for organ chips [90]. Although, the platform has been increasingly used for studying the initiation and progression of infectious diseases, culture time of organ chips are currently limited to weeks and are not suitable for the study of long-term effects in which a disease may progress for years, such as in chronic hepatitis. Adoption of organ-on-chip technologies is also limited due to the technical knowledge required for device design and the lack of integration with available high-throughput screening systems [91]. In terms of the validation and quality control of organ chips, concerns still exist that require universal device design and device fabrication and extensive testing to prove robustness and reproducibility before reaching a translational stage to enable specific regulations and standardization for greater adoption of the devices [92]. Finally, organomimetic devices usually require operational equipment that apply mechanical forces to the cells and the flow of fluid through the microfluidic channels, making it difficult to adapt and expensive to set-up [93]. 

## 8. Conclusions

Although still at its infancy, so far, the studies on organ chips have illustrated the advantages of this technology over existing in vitro models. With the growing ethical concerns on the use of animals in biomedical research along with the limitations of standard tissue culture methods and in vivo models, a considerable shift to the use of patient and/or organ-specific chips in research is expected. Enabling accurate reconstitution, direct observation, and quantitative analysis of organ-level physiological functions, organ chips may in the future be supported by 3D printing technology for a rapid fabrication. Throughput analysis of available organ chips can be improved by expanding the currently used high-content microscopy imaging to alternative screening assays such as CRISPR/Cas9 functional genomic screens, single-cell sequencing, and metabolomics platforms. Through its applications in major areas—such as bacterial and viral evolution, transmission dynamics, and drug screening during pandemics—organ-on-chip technology is expected to be at the forefront of infectious disease research in the near future.

## References

[1] Sin A., Chin K.C., Jamil M.F., Kostov Y., Rao G., Shuler M.L. (2004). The Design and Fabrication of Three-Chamber Microscale Cell Culture Analog Devices with Integrated Dissolved Oxygen Sensors. Biotechnol. Prog..

[2] Viravaidya K., Sin A., Shuler M.L. (2004). Development of a Microscale Cell Culture Analog to Probe Naphthalene Toxicity. Biotechnol. Prog..

[3] Huh D., Matthews B.D., Mammoto A., Montoya-Zavala M., Yuan Hsin H., Ingber D.E. (2010). Reconstituting organ-level lung functions on a chip. Science.

[4] Huh D., Fujioka H., Tung Y.C., Futai N., Paine R., Grotberg J.B., Takayama S. (2007). Acoustically detectable cellular-level lung injury induced by fluid mechanical stresses in microfluidic airway systems. Proc. Natl. Acad. Sci. USA.

[5] Huh D., Kim H.J., Fraser J.P., Shea D.E., Khan M., Bahinski A., Hamilton G.A., Ingber D.E. (2013). Microfabrication of human organs-on-chips. Nat. Protoc..

[6] Mahler G.J., Esch M.B., Glahn R.P., Shuler M.L. (2009). Characterization of a gastrointestinal tract microscale cell culture analog used to predict drug toxicity. Biotechnol. Bioeng..

[7] Kimura H., Yamamoto T., Sakai H., Sakai Y., Fujii T. (2008). An integrated microfluidic system for long-term perfusion culture and on-line monitoring of intestinal tissue models. Lab Chip.

[8] Lee P.J., Hung P.J., Lee L.P. (2007). An artificial liver sinusoid with a microfluidic endothelial-like barrier for primary hepatocyte culture. Biotechnol. Bioeng..

[9] Carraro A., Hsu W.M., Kulig K.M., Cheung W.S., Miller M.L., Weinberg E.J., Swart E.F., Kaazempur-Mofrad M., Borenstein J.T., Vacanti J.P. (2008). In Vitro analysis of a hepatic device with intrinsic microvascular-based channels. Biomed. Microdevices.

[10] Jang K.J., Suh K.Y. (2010). A multi-layer microfluidic device for efficient culture and analysis of renal tubular cells. Lab Chip.

[11] Harris S.G., Shuler M.L. (2003). Growth of endothelial cells on microfabricated silicon nitride membranes for an In Vitro model of the blood-brain barrier. Biotechnol. Bioprocess Eng..

[12] Booth R., Kim H. (2012). Characterization of a microfluidic in vitro model of the blood-brain barrier (μBBB). Lab Chip.

[13] Agarwal A., Goss J.A., Cho A., McCain M.L., Parker K.K. (2013). Microfluidic heart on a chip for higher throughput pharmacological studies. Lab Chip.

[14] Hou L., Hagen J., Wang X., Papautsky I., Naik R., Kelley-Loughnane N., Heikenfeld J. (2013). Artificial microfluidic skin for in vitro perspiration simulation and testing. Lab Chip.

[15] Song J.W., Gu W., Futai N., Warner K.A., Nor J.E., Takayama S. (2005). Computer-controlled microcirculatory support system for endothelial cell culture and shearing. Anal. Chem..

[16] Jang K., Sato K., Igawa K., Chung U.-I., Kitamori T. (2008). Development of an osteoblast-based 3D continuous-perfusion microfluidic system for drug screening. Anal. Bioanal. Chem..

[17] Liu Y., Kongsuphol P., Gourikutty S.B.N., Ramadan Q. (2017). Human adipocyte differentiation and characterization in a perfusion-based cell culture device. Biomed. Microdevices.

[18] Lee J.S., Romero R., Han Y.M., Kim H.C., Kim C.J., Hong J.S., Huh D. (2016). Placenta-on-A-chip: A novel platform to study the biology of the human placenta. J. Matern. Fetal Neonatal Med..

[19] Zervantonakis I.K., Hughes-Alford S.K., Charest J.L., Condeelis J.S., Gertler F.B., Kamm R.D. (2012). Three-dimensional microfluidic model for tumor cell intravasation and endothelial barrier function. Proc. Natl. Acad. Sci. USA.

[20] Shirure V.S., Bi Y., Curtis M.B., Lezia A., Goedegebuure M.M., Goedegebuure S.P., Aft R., Fields R.C., George S.C. (2018). Tumor-on-a-chip platform to investigate progression and drug sensitivity in cell lines and patient-derived organoids. Lab Chip.

[21] Zhang Y.S., Davoudi F., Walch P., Manbachi A., Luo X., Dell’Erba V., Miri A.K., Albadawi H., Arneri A., Li X. (2016). Bioprinted thrombosis-on-a-chip. Lab Chip.

[22] Park J., Lee B.K., Jeong G.S., Hyun J.K., Lee C.J., Lee S.H. (2015). Three-dimensional brain-on-a-chip with an interstitial level of flow and its application as an in vitro model of Alzheimer’s disease. Lab Chip.

[23] Zhu Y., Warrick J.W., Haubert K., Beebe D.J., Yin J. (2009). Infection on a chip: A microscale platform for simple and sensitive cell-based virus assays. Biomed. Microdevices.

[24] Wu Q., Liu J., Wang X., Feng L., Wu J., Zhu X., Wen W., Gong X. (2020). Organ-on-a-chip: Recent breakthroughs and future prospects. Biomed. Eng. Online.

[25] Ergir E., Bachmann B., Redl H., Forte G., Ertl P. (2018). Small force, big impact: Next generation organ-on-a-chip systems incorporating biomechanical cues. Front. Physiol..

[26] Zhang B., Korolj A., Lai B.F.L., Radisic M. (2018). Advances in organ-on-a-chip engineering. Nat. Rev. Mater..

[27] Sosa-Hernández J.E., Villalba-Rodríguez A.M., Romero-Castillo K.D., Aguilar-Aguila-Isaías M.A., García-Reyes I.E., Hernández-Antonio A., Ahmed I., Sharma A., Parra-Saldívar R., Iqbal H.M.N. (2018). Organs-on-a-chip module: A review from the development and applications perspective. Micromachines.

[28] Yi H.G., Lee H., Cho D.W. (2017). 3D printing of organs-on-chips. Bioengineering.

[29] Barrila J., Crabbé A., Yang J., Franco K., Nydam S.D., Forsyth R.J., Davis R.R., Gangaraju S., Mark Ott C., Coyne C.B. (2018). Modeling host-pathogen interactions in the context of the microenvironment: Three-dimensional cell culture comes of age. Infect. Immun..

[30] Ingber D.E. (2020). Is it Time for Reviewer 3 to Request Human Organ Chip Experiments Instead of Animal Validation Studies?. Adv. Sci..

[31] Fasciano A.C., Mecsas J., Isberg R.R. (2019). New Age Strategies To Reconstruct Mucosal Tissue Colonization and Growth in Cell Culture Systems. Microbiol. Spectr..

[32] Mounier J., Vasselon T., Hellio R., Lesourd M., Sansonetti P.J. (1992). Shigella flexneri enters human colonic Caco-2 epithelial cells through the basolateral pole. Infect. Immun..

[33] Koestler B.J., Ward C.M., Fisher C.R., Rajan A., Maresso A.W., Payne S.M. (2019). Human intestinal enteroids as a model system of shigella pathogenesis. Infect. Immun..

[34] Ranganathan S., Doucet M., Grassel C.L., Delaine-Elias B.O., Zachos N.C., Barry E.M. (2019). Evaluating shigella flexneri pathogenesis in the human enteroid model. Infect. Immun..

[35] Kasendra M., Tovaglieri A., Sontheimer-Phelps A., Jalili-Firoozinezhad S., Bein A., Chalkiadaki A., Scholl W., Zhang C., Rickner H., Richmond C.A. (2018). Development of a primary human Small Intestine-on-a-Chip using biopsy-derived organoids. Sci. Rep..

[36] Jalili-Firoozinezhad S., Prantil-Baun R., Jiang A., Potla R., Mammoto T., Weaver J.C., Ferrante T.C., Kim H.J., Cabral J.M.S., Levy O. (2018). Modeling radiation injury-induced cell death and countermeasure drug responses in a human Gut-on-a-Chip article. Cell Death Dis..

[37] Guo Y., Li Z., Su W., Wang L., Zhu Y., Qin J. (2018). A Biomimetic Human Gut-on-a-Chip for Modeling Drug Metabolism in Intestine. Artif. Organs.

[38] Grassart A., Malardé V., Gobba S., Sartori-Rupp A., Kerns J., Karalis K., Marteyn B., Sansonetti P., Sauvonnet N. (2019). Bioengineered Human Organ-on-Chip Reveals Intestinal Microenvironment and Mechanical Forces Impacting Shigella Infection. Cell Host Microbe.

[39] Sunuwar L., Yin J., Kasendra M., Karalis K., Kaper J., Fleckenstein J., Donowitz M. (2020). Mechanical stimuli affect Escherichia coli heat-stable enterotoxin-cyclic GMP signaling in a human enteroid intestine-chip model. Infect. Immun..

[40] Torrelles J.B., Schlesinger L.S. (2017). Integrating Lung Physiology, Immunology, and Tuberculosis. Trends Microbiol..

[41] Thacker V.V., Dhar N., Sharma K., Barrile R., Karalis K., McKinney J.D. (2020). A lung-on-chip model of early M. tuberculosis infection reveals an essential role for alveolar epithelial cells in controlling bacterial growth. eLife.

[42] Deinhardt-Emmer S., Rennert K., Schicke E., Cseresnyés Z., Windolph M., Nietzsche S., Heller R., Siwczak F., Haupt K.F., Carlstedt S. (2020). Co-infection with Staphylococcus aureus after primary influenza virus infection leads to damage of the endothelium in a human alveolus-on-a-chip model. Biofabrication.

[43] Shi D., Mi G., Wang M., Webster T.J. (2019). In Vitro and Ex Vivo systems at the forefront of infection modeling and drug discovery. Biomaterials.

[44] Terry J., Neethirajan S. (2014). A novel microfluidic wound model for testing antimicrobial agents against Staphylococcus pseudintermedius biofilms. J. Nanobiotechnol..

[45] Wright E., Neethirajan S., Weng X. (2015). Microfluidic wound model for studying the behaviors of Pseudomonas aeruginosa in polymicrobial biofilms. Biotechnol. Bioeng..

[46] Tang H., Abouleila Y., Si L., Ortega-Prieto A.M., Mummery C.L., Ingber D.E., Mashaghi A. (2020). Human Organs-on-Chips for Virology. Trends Microbiol..

[47] Benam K.H., Villenave R., Lucchesi C., Varone A., Hubeau C., Lee H.H., Alves S.E., Salmon M., Ferrante T.C., Weaver J.C. (2016). Small airway-on-a-chip enables analysis of human lung inflammation and drug responses in vitro. Nat. Methods.

[48] Ortega-Prieto A.M., Cherry C., Gunn H., Dorner M. (2019). In Vivo Model Systems for Hepatitis B Virus Research. ACS Infect. Dis..

[49] Kang Y.B.A., Sodunke T.R., Lamontagne J., Cirillo J., Rajiv C., Bouchard M.J., Noh M. (2015). Liver sinusoid on a chip: Long-term layered co-culture of primary rat hepatocytes and endothelial cells in microfluidic platforms. Biotechnol. Bioeng..

[50] Kang Y.B., Rawat S., Duchemin N., Bouchard M., Noh M. (2017). Human liver sinusoid on a chip for hepatitis B virus replication study. Micromachines.

[51] Ortega-Prieto A.M., Skelton J.K., Wai S.N., Large E., Lussignol M., Vizcay-Barrena G., Hughes D., Fleck R.A., Thursz M., Catanese M.T. (2018). 3D microfluidic liver cultures as a physiological preclinical tool for hepatitis B virus infection. Nat. Commun..

[52] Villenave R., Wales S.Q., Hamkins-Indik T., Papafragkou E., Weaver J.C., Ferrante T.C., Bahinski A., Elkins C.A., Kulka M., Ingber D.E. (2017). Human gut-on-a-chip supports polarized infection of coxsackie B1 virus in vitro. PLoS ONE.

[53] Miccoli B., Braeken D., Li Y.-C.E. (2019). Brain-on-a-chip Devices for Drug Screening and Disease Modeling Applications. Curr. Pharm. Des..

[54] Johnson B.N., Lancaster K.Z., Hogue I.B., Meng F., Kong Y.L., Enquist L.W., McAlpine M.C. (2016). 3D printed nervous system on a chip. Lab Chip.

[55] Wang J., Wang C., Xu N., Liu Z.-F., Pang D.-W., Zhang Z.-L. (2019). A virus-induced kidney disease model based on organ-on-a-chip: Pathogenesis exploration of virus-related renal dysfunctions. Biomaterials.

[56] Nawroth J.C., Lucchesi C., Cheng D., Shukla A., Ngyuen J., Shroff T., Varone A., Karalis K., Lee H.H., Alves S. (2020). A microengineered airway lung chip models key features of viral-induced exacerbation of asthma. Am. J. Respir. Cell Mol. Biol..

[57] Si L., Prantil-Baun R., Benam K.H., Bai H., Rodas M., Burt M., Ingber D.E. (2019). Discovery of influenza drug resistance mutations and host therapeutic targets using a human airway chip. bioRxiv.

[58] Steel J., Lowen A.C. (2014). Influenza a virus reassortment. Curr. Top. Microbiol. Immunol..

[59] Si L., Bai H., Rodas M., Cao W., Oh C.Y., Jiang A., Nurani A., Zhu D., Goyal G., Gilpin S. (2020). Human organs-on-chips as tools for repurposing approved drugs as potential influenza and COVID19 therapeutics in viral pandemics. bioRxiv.

[60] Zhang M., Wang P., Luo R., Wang Y., Li Z., Guo Y., Yao Y., Li M., Tao T., Chen W. (2020). Biomimetic Human Disease Model of SARS-CoV-2 Induced Lung Injury and Immune Responses on Organ Chip System. Adv. Sci..

[61] Junaid A., Tang H., van Reeuwijk A., Abouleila Y., Wuelfroth P., van Duinen V., Stam W., van Zonneveld A.J., Hankemeier T., Mashaghi A. (2020). Ebola Hemorrhagic Shock Syndrome-on-a-Chip. iScience.

[62] Henry B., Roussel C., Carucci M., Brousse V., Ndour P.A., Buffet P. (2020). The Human Spleen in Malaria: Filter or Shelter?. Trends Parasitol..

[63] Rigat-Brugarolas L.G., Elizalde-Torrent A., Bernabeu M., De Niz M., Martin-Jaular L., Fernandez-Becerra C., Homs-Corbera A., Samitier J., Del Portillo H.A. (2014). A functional microengineered model of the human splenon-on-a-chip. Lab Chip.

[64] Introini V., Carciati A., Tomaiuolo G., Cicuta P., Guido S. (2018). Endothelial glycocalyx regulates cytoadherence in Plasmodium falciparum malaria. J. R. Soc. Interface.

[65] Arakawa C., Gunnarsson C., Howard C., Bernabeu M., Phong K., Yang E., DeForest C.A., Smith J.D., Zheng Y. (2020). Biophysical and biomolecular interactions of malaria-infected erythrocytes in engineered human capillaries. Sci. Adv..

[66] Picot J., Ndour P.A., Lefevre S.D., El Nemer W., Tawfik H., Galimand J., Da Costa L., Ribeil J.A., de Montalembert M., Brousse V. (2015). A biomimetic microfluidic chip to study the circulation and mechanical retention of red blood cells in the spleen. Am. J. Hematol..

[67] Burton A. (2017). Gaining ground against cerebral malaria. Lancet Neurol..

[68] Baydoun M., Vanneste S.B., Creusy C., Guyot K., Gantois N., Chabe M., Delaire B., Mouray A., Baydoun A., Forzy G. (2017). Three-dimensional (3D) culture of adult murine colon as an in vitro model of cryptosporidiosis: Proof of concept. Sci. Rep..

[69] Gunasekera S., Zahedi A., O’dea M., King B., Monis P., Thierry B., Carr J.M., Ryan U. (2020). Organoids and bioengineered intestinal models: Potential solutions to the Cryptosporidium culturing dilemma. Microorganisms.

[70] Duque-Correa M.A., Maizels R.M., Grencis R.K., Berriman M. (2020). Organoids—New Models for Host–Helminth Interactions. Trends Parasitol..

[71] Asghar W., Sher M., Khan N.S., Vyas J.M., Demirci U. (2019). Microfluidic Chip for Detection of Fungal Infections. ACS Omega.

[72] Qiang L., Guo J., Han Y., Jiang J., Su X., Liu H., Qi Q., Han L. (2019). A novel anti Candida albicans drug screening system based on high-throughput microfluidic chips. Sci. Rep..

[73] Barkal L.J., Walsh N.M., Botts M.R., Beebe D.J., Hull C.M. (2016). Leveraging a high resolution microfluidic assay reveals insights into pathogenic fungal spore germination. Integr. Biol..

[74] May S., Evans S., Parry L. (2017). Organoids, organs-on-chips and other systems, and microbiota. Emerg. Top. Life Sci..

[75] Kim H.J., Huh D., Hamilton G., Ingber D.E. (2012). Human gut-on-a-chip inhabited by microbial flora that experiences intestinal peristalsis-like motions and flow. Lab Chip.

[76] Dai C., Zhao D.H., Jiang M. (2012). VSL#3 probiotics regulate the intestinal epithelial barrier in Vivo and in Vitro via the p38 and ERK signaling pathways. Int. J. Mol. Med..

[77] Kim H.J., Li H., Collins J.J., Ingber D.E. (2016). Contributions of microbiome and mechanical deformation to intestinal bacterial overgrowth and inflammation in a human gut-on-a-chip. Proc. Natl. Acad. Sci. USA.

[78] Shin W., Kim H.J. (2018). Intestinal barrier dysfunction orchestrates the onset of inflammatory host-microbiome cross-talk in a human gut inflammation-on-a-chip. Proc. Natl. Acad. Sci. USA.

[79] Fukuda S., Toh H., Hase K., Oshima K., Nakanishi Y., Yoshimura K., Tobe T., Clarke J.M., Topping D.L., Suzuki T. (2011). Bifidobacteria can protect from enteropathogenic infection through production of acetate. Nature.

[80] Jacobson A., Lam L., Rajendram M., Tamburini F., Honeycutt J., Pham T., Van Treuren W., Pruss K., Stabler S.R., Lugo K. (2018). A Gut Commensal-Produced Metabolite Mediates Colonization Resistance to Salmonella Infection. Cell Host Microbe.

[81] Tovaglieri A., Sontheimer-Phelps A., Geirnaert A., Prantil-Baun R., Camacho D.M., Chou D.B., Jalili-Firoozinezhad S., De Wouters T., Kasendra M., Super M. (2019). Species-specific enhancement of enterohemorrhagic E. coli pathogenesis mediated by microbiome metabolites. Microbiome.

[82] Ramadan Q., Jafarpoorchekab H., Huang C., Silacci P., Carrara S., Koklü G., Ghaye J., Ramsden J., Ruffert C., Vergeres G. (2013). NutriChip: Nutrition analysis meets microfluidics. Lab Chip.

[83] Shah P., Fritz J.V., Glaab E., Desai M.S., Greenhalgh K., Frachet A., Niegowska M., Estes M., Jäger C., Seguin-Devaux C. (2016). A microfluidics-based in vitro model of the gastrointestinal human-microbe interface. Nat. Commun..

[84] Trujillo-de Santiago G., Lobo-Zegers M.J., Montes-Fonseca S.L., Zhang Y.S., Alvarez M.M. (2018). Gut-microbiota-on-a-chip: An enabling field for physiological research. Microphysiol. Syst..

[85] Barr J.J., Auro R., Sam-Soon N., Kassegne S., Peters G., Bonilla N., Hatay M., Mourtada S., Bailey B., Youle M. (2015). Subdiffusive motion of bacteriophage in mucosal surfaces increases the frequency of bacterial encounters. Proc. Natl. Acad. Sci. USA.

[86] Barr J.J. (2019). Missing a Phage: Unraveling Tripartite Symbioses within the Human Gut. mSystems.

[87] Ingber D.E. (2018). Developmentally inspired human ‘organs on chips’. Development.

[88] Wong I., Ho C.M. (2009). Surface molecular property modifications for poly(dimethylsiloxane) (PDMS) based microfluidic devices. Microfluid. Nanofluid..

[89] Fritschen A., Blaeser A. (2021). Biosynthetic, biomimetic, and self-assembled vascularized Organ-on-a-Chip systems. Biomaterials.

[90] Quan Y., Sun M., Tan Z., Eijkel J.C.T., Van Den Berg A., Van Der Meer A., Xie Y. (2020). Organ-on-a-chip: The next generation platform for risk assessment of radiobiology. RSC Adv..

[91] Wnorowski A., Yang H., Wu J.C. (2019). Progress, obstacles, and limitations in the use of stem cells in organ-on-a-chip models. Adv. Drug Deliv. Rev..

[92] Allwardt V., Ainscough A.J., Viswanathan P., Sherrod S.D., McLean J.A., Haddrick M., Pensabene V. (2020). Translational roadmap for the organs-on-a-chip industry toward broad adoption. Bioengineering.

[93] Bassi G., Grimaudo M.A., Panseri S., Montesi M. (2021). Advanced multi-dimensional cellular models as emerging reality to reproduce In Vitro the human body complexity. Int. J. Mol. Sci..

